# Impact of Adherence to Guideline‐Directed Prevention Strategies on Clinical Outcomes in Patients With Coronary Artery Disease and Diabetes Mellitus Following Acute Coronary Syndrome: A 3‐Year Cohort Study

**DOI:** 10.1002/clc.70164

**Published:** 2025-06-02

**Authors:** Nur Kamer Kaya İnalkaç, Fuat Polat, İbrahim Keleş

**Affiliations:** ^1^ Department of Cardiology, İstanbul University‐Cerrahpasa Cerrahpaşa Faculty of Medicine İstanbul Türkiye; ^2^ Department of Cardiology Dr. Siyami Ersek Thoracic and Cardiovascular Surgery Educatıon Research Hospıtal İstanbul Türkiye; ^3^ University of Health Sciences İstanbul Türkiye

**Keywords:** coronary artery disease, mortality, prevention strategies, rehospitalization, type 2 diabetes mellitus

## Abstract

**Background:**

Coronary artery disease (CAD) and diabetes mellitus (DM) significantly increase the risk after acute coronary syndrome. This study evaluated adherence to guideline‐directed secondary prevention strategies and demonstrated their substantial impact on reducing rehospitalization and mortality in this population.

**Methods:**

A retrospective cohort study was conducted on 987 CAD and DM patients admitted for ACS between 2015 and 2018. Adherence to seven evidence‐based secondary prevention strategies was assessed: smoking cessation, physical activity, antiplatelet therapy, statins, blood pressure control, ACEi/ARB therapy, and SGLT‐2i therapy. Patients were categorized into groups based on the number of recommendations followed (0–2, 3–4, and 5+). Primary outcomes included rehospitalization and all‐cause mortality over a 3‐year follow‐up period.

**Results:**

At baseline, only 12.4% of patients adhered to five or more recommendations, which dramatically increased to 71.9% by the 3‐year follow‐up. Individual adherence to each of blood pressure control (HR = 0.81, 95% CI: 0.70–0.94), ACEi/ARB therapy (HR = 0.77, 95% CI: 0.67–0.89), and SGLT‐2i therapy (HR = 0.79, 95% CI: 0.68–0.92) significantly reduced rehospitalization risk. Similarly, adherence to these therapies individually reduced mortality risk (HR = 0.78, 95% CI: 0.67–0.91; HR = 0.74, 95% CI: 0.63–0.87; and HR = 0.72, 95% CI: 0.61–0.85, respectively). Importantly, a stepwise increase in adherence was associated with a dose‐dependent reduction in mortality (HR = 0.65, 95% CI: 0.52–0.81, *p* < 0.05).

**Conclusion:**

This study highlights the critical role of comprehensive, multifactorial secondary prevention in its association with improved long‐term outcomes in patients with CAD and DM following ACS.

## Graphical abstract

In patients with coronary artery disease and diabetes following acute coronary syndrome, adherence to five or more guideline‐directed prevention strategies resulted in a 35% reduction in mortality compared to limited adherence, highlighting the dose‐dependent relationship between comprehensive preventive care and improved clinical outcomes.

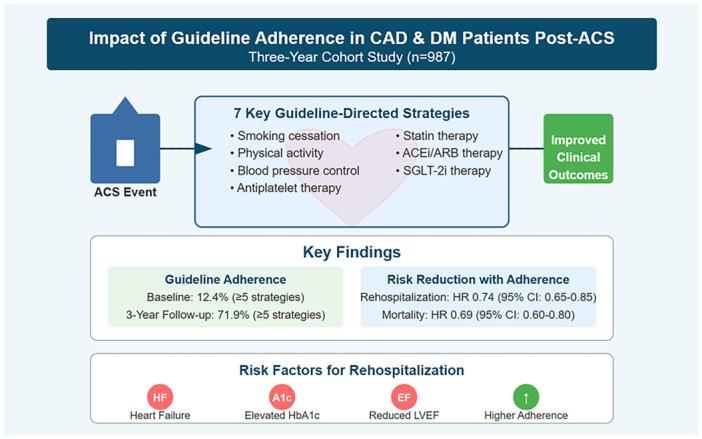

## 1 Introduction

Coronary artery disease (CAD) is a leading cause of morbidity and mortality worldwide, particularly when complicated by type 2 diabetes mellitus (DM) [1, 2, 3]. The presence of both conditions significantly increases the risk of acute coronary syndrome (ACS) and worsens clinical outcomes [4]. ACS encompasses a range of conditions, including ST‐elevation myocardial infarction (STEMI), non‐ST‐elevation myocardial infarction (NSTEMI), and unstable angina pectoris (UAP), all of which contribute to the burden of CAD. Effective management of ACS in patients with CAD and DM relies on comprehensive secondary prevention strategies to reduce the risk of recurrent events, rehospitalization, and mortality [5, 6].

The implementation of guideline‐directed recommendations for secondary prevention has been shown to improve outcomes in patients with ACS [7, 8, 9]. Primary prevention strategies include smoking cessation and maintaining physical activity, while secondary prevention focuses on controlling risk factors such as antiplatelet therapy, statin use, blood pressure regulation, and the use of angiotensin‐converting enzyme inhibitors/angiotensin receptor blockers (ACEi/ARB) and sodium–glucose cotransporter 2 inhibitors (SGLT‐2i). Notably, statin therapy has also been shown to improve outcomes in patients with stable CAD undergoing percutaneous coronary intervention (PCI), further emphasizing its critical role in cardiovascular risk reduction [10]. Despite the known benefits of these interventions, adherence to recommended treatment regimens remains suboptimal in real‐world clinical settings [11, 12].

This study aimed to evaluate adherence to seven guideline‐based secondary prevention recommendations in patients with CAD and DM presenting with ACS. The adherence rates were assessed before and 3 years after the index ACS. In addition, the impact of adherence to these recommendations on rehospitalization and mortality was analyzed, with a focus on identifying demographic and clinical factors associated with these outcomes.

The objectives of this study were to assess the frequency of adherence to primary and secondary prevention strategies, evaluate the clinical outcomes associated with adherence, and identify factors that may influence rehospitalization and mortality in this patient population. We hypothesized that [1]: adherence to ≥ 5 guideline‐directed secondary prevention strategies would be associated with significantly lower rates of all‐cause mortality compared to adherence to ≤ 4 strategies (HR < 0.70) [2]; individual adherence to statins, ACEi/ARBs, and SGLT‐2 inhibitors would demonstrate the strongest independent associations with reduced mortality; and [3] the mortality benefit would be most pronounced in patients with both optimal adherence (≥ 5 strategies) and controlled HbA1c (< 7.0%).

## 2 Methods

### 2.1 Study Design and Population

This retrospective cohort study was conducted at a tertiary care center and included patients with confirmed CAD and type 2 DM who presented with ACS between September 2019 and December 2022. This design was specifically selected to enable efficient evaluation of the association between adherence to guideline‐directed prevention strategies (exposure) and critical clinical outcomes (rehospitalization and mortality). Given the significant public health impact of suboptimal secondary prevention in this high‐risk population, a retrospective approach allowed for the timely generation of evidence to inform clinical practice improvements, which would not have been feasible with a multiyear prospective design. Additionally, the retrospective methodology facilitated the assessment of multiple exposures (seven distinct prevention strategies) simultaneously, providing comprehensive insights into their relative contributions to patient outcomes.

A retrospective cohort design was specifically selected not only to efficiently evaluate the association between adherence to guideline‐directed prevention strategies and critical clinical outcomes, but also because this approach is particularly well‐suited for studying the cumulative effects of multiple exposures over an extended period. This design offers distinct advantages when investigating outcomes with potentially long latency periods following exposures, such as the delayed clinical benefits of preventive interventions in cardiovascular disease. Additionally, the retrospective methodology allowed us to assess multiple exposures (seven distinct prevention strategies) simultaneously with sufficient statistical power, providing comprehensive insights into their relative contributions to patient outcomes that would have been challenging to achieve through alternative study designs requiring prospective recruitment of a comparable high‐risk population.

Ethics committee approval was obtained prior to study initiation. Patients were identified through systematic screening of the hospital's electronic health record system using ICD‐10 codes for ACS (I21.0–I21.9, I22.0–I22.9) and type 2 DM (E11.0–E11.9). Eligible patients were further verified through manual chart review by two independent investigators to confirm both the diagnosis of CAD (based on coronary angiography demonstrating ≥ 50% stenosis in at least one major epicardial coronary artery) and acute ACS (confirmed by a combination of clinical presentation, cardiac biomarker elevations, and characteristic ECG changes in accordance with the Fourth Universal Definition of Myocardial Infarction). Consecutive patients meeting the inclusion criteria were enrolled to minimize selection bias. The study aimed to evaluate adherence to seven guideline‐based secondary prevention recommendations and analyze their impact on rehospitalization and mortality over a 3‐year follow‐up period.

### 2.2 Inclusion and Exclusion Criteria

**Figure 1:**
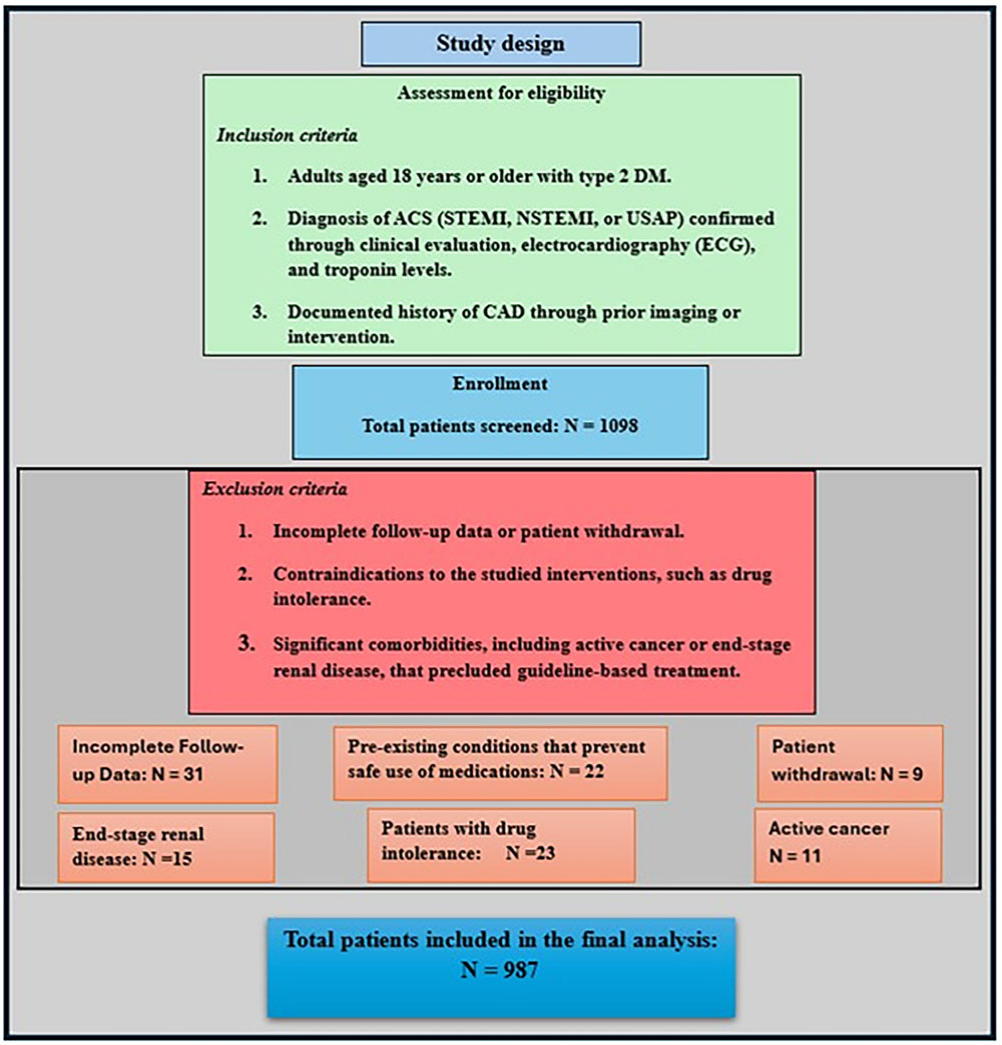
Study flowchart.

Key inclusion criteria were: age ≥ 18 years; confirmed CAD based on coronary angiography; established type 2 DM; presentation with ACS during the study period; at least one documented medical encounter within 12 months prior to the index ACS event; and minimum 36 months of follow‐up data. Key exclusion criteria were: life expectancy < 1 year due to noncardiovascular causes; type 1 DM; end‐stage kidney disease; and severe hepatic dysfunction. The full list of inclusion and exclusion criteria is provided in Appendix S1: Supplementary Methods, and a study flowchart is provided in Figure 1.

### 2.3 Data Collection

Data were extracted from electronic health records and included demographic characteristics, clinical variables, and adherence rates to seven guideline‐based recommendations. Baseline characteristics included age, gender, HbA1c levels, hypertension, and prior revascularization. Echocardiographic parameters, including left ventricular ejection fraction (LVEF), were recorded within the first 24 h of the index ACS.

### 2.4 Guideline‐Recommended Prevention Strategies

Seven guideline‐based secondary prevention strategies were evaluated:

1. Smoking cessation/nonsmoking
2. Physical activity maintenance
3. Antiplatelet therapy
4. Statin therapy
5. Blood pressure control
6. ACE inhibitors (ACEi)/angiotensin receptor blockers (ARBs) therapy
7. Sodium–glucose cotransporter 2 inhibitors (SGLT‐2i) therapy

Detailed criteria for adherence classification, including standardized definitions for each preventive measure, criteria for appropriate versus inappropriate medication nonuse, and methods for distinguishing between contraindications, intolerance, and true nonadherence, are provided in Appendix S1: Supplementary Methods.

Patients were categorized into three adherence groups based on the number of recommendations followed:

- Group 1: adherence to ≤ 2 recommendations.
- Group 2: adherence to 3–4 recommendations.
- Group 3: adherence to ≥ 5 recommendations.

### 2.5 Outcomes and Follow‐Up

The primary outcomes were rehospitalization and all‐cause mortality over a 3‐year follow‐up period. Rehospitalization events were confirmed through hospital records, and mortality data were verified using national death registries and death certificates to determine the cause of death when available.

Secondary outcomes included the association of demographic and clinical factors (e.g., heart failure, HbA1c levels, and LVEF) with rehospitalization and mortality and the effectiveness of specific prevention measures in reducing these outcomes.

### 2.6 Statistical Analysis

Descriptive statistics were used to summarize baseline characteristics, adherence rates, and outcome frequencies. Continuous variables were expressed as mean ± standard deviation (SD), and categorical variables were presented as frequencies and percentages. Paired *t*‐tests were used to evaluate changes in adherence rates over time.

The impact of demographic and clinical factors on rehospitalization and mortality was assessed using multivariate Cox regression analysis, with results expressed as hazard ratios (HR) and 95% confidence intervals (CI). Variables for inclusion in the multivariable model were selected based on a combination of clinical relevance, statistical significance in univariate analysis (*p* < 0.10), and established risk factors from prior literature. Specifically, we included demographic factors (age, gender), clinical parameters (HbA1c, LVEF, heart failure status, anemia), and adherence to each of the seven prevention strategies. A stepwise selection approach with entry criterion of *p* < 0.10 and stay criterion of *p* < 0.05 was used to determine the final model. Log‐rank tests were employed to compare survival curves across adherence groups. Kaplan–Meier survival analysis was used to visualize survival differences between adherence categories.

Multiple regression analysis was used to evaluate differences in rehospitalization and mortality between adherence categories. Statistical significance was set at a *p* < 0.05, and all analyses were performed using SPSS software version 25.0.

## 3 Results

A total of 987 patients with CAD and type 2 DM presenting with ACS were included in the study. The mean age was 59.3 ± 10.9 years, and 61.9% were male. The mean HbA1c level was 8.8 ± 2.0, with 61.6% of patients having a history of hypertension. Prior revascularization, either through percutaneous intervention or coronary artery bypass grafting, was noted in 35.2% of the cohort. Echocardiographic evaluation within the first 24 h of ACS revealed a mean LVEF of 54.2% ± 11.6%. The index diagnosis included STEMI in 53%, NSTEMI in 43.5%, and UAP in 3.5% of patients (Table 1).

**Table 1:** Clinical and demographic characteristics of the study population.

Patient characteristics	*N* = 987
Age (year)	59.3 ± 10.9
Gender (male) %	61.9 (611)
BMI (kg/m^2^)	29.2 ± 4.0
History of CAD %	100.0 (987)
DM %	100.0 (987)
HT %	61.6 (608)
Dislipidemia %	37.1 (366)
History of PCI %	27.2 (268)
History of CABG %	8.0 (79)
CRF %	13.5 (133)
HF %	43.7 (431)
CVA %	11.1 (110)
PAD %	6.4 (63)
COPD %	3.2 [32]
LVEF (%)	54.2 ± 11.6
Type of ACS	
STEMI %	53.0 (523)
NSTEMI %	43.5 (429)
UAP %	3.5 [35]
eGFR (mL/min/1.73 m^2^)	88.6.2 ± 31.6
Hemoglobin (mg/dL)	12.3 ± 3.2
HbA1c (%)	8.8 ± 2.0
Heart rate (bpm)	83.0 ± 16.2

*Note:* Continuous variables are given as mean ± SD. Median, interquartile range (range, [25% percentile–75% percentile]).

Abbreviations: ACS, acute coronary syndrome; BMI, body mass index; CABG, coronary artery bypass grafting; CAD, coronary artery disease; COPD, chronic obstructive pulmonary disease; CRF, chronic renal failure; CVA, cerebral vascular accident; DM, diabetes mellitus; GFR, glomerular filtration rate; HbA1c, hemoglobin A1c; HF, heart failure; HT, hypertension; LVEF, left ventricular ejection fraction; NSTEMI, non‐ST‐elevation myocardial infarction; PAD, peripheric artery disease; PCI, percutaneous intervention; STEMI, ST‐elevation myocardial infarction; WBC, white blood cell; UAP, unstable angina pectoris.

Prior to the index ACS, 37.4% of patients were not on antiplatelet therapy, which significantly decreased to 6.6% by the 3rd‐year follow‐up (*p* < 0.001). Statin use increased from 19.3% before ACS to 79.6% during the same period (*p* < 0.001). Blood pressure control improved significantly, rising from 29.8% before ACS to 89.7% at the 3rd‐year follow‐up (*p* < 0.001). The use of ACE inhibitors or ARBs was assessed only in patients with hypertension or relevant indications for these medications, and adherence to these therapies increased from 36.8% to 73.6% (*p* < 0.001). SGLT‐2 inhibitor utilization rose from 32.4% to 44.0% (*p* < 0.001). The proportion of nonsmokers increased from 47.8% to 54.6% (*p* = 0.002). Physical activity levels remained consistent, with 68.6% of patients reporting at least moderate activity before ACS compared to 71.3% at the 3rd‐year follow‐up (*p* = 0.182) (Table 2).

**Table 2:** Adherence to secondary and primary prevention guidelines in patients with coronary artery disease and diabetes mellitus before and 3 years after acute coronary syndrome.

Patients characteristics	Before acute coronary syndrome	After acute coronary syndrome	Overall *p* value
Antiplatelet	62.6 (618)	93.4 (922)	< 0.001
Statin	19.3 (190)	79.6 (786)	< 0.001
Blood pressure regulation	29.8 (294)	89.7 (885)	< 0.001
ACEi/ARB treatment	36.8 (363)	73.6 (726)	< 0.001
SGLT‐2i treatment	32.4 (320)	44.0 (434)	< 0.001
Not using/quitting smoking	47.8 (472)	54.6 (539)	0.002
Physically active	68.6 (677)	71.3 (704)	0.182

*Note:* Values are in the form of % (*n*).

Abbreviations: ACEi/ARB, angiotensin‐converting enzyme inhibitors/angiotensin II receptor blockers; SGLT‐2i, sodium–glucose cotransporter‐2.

Adherence to seven secondary prevention strategies—comprising smoking cessation or nonsmoking, maintaining physical activity, antiplatelet therapy, statin use, blood pressure regulation, ACEi/ARB therapy, and SGLT‐2i therapy—was assessed in patients with CAD and DM. This evaluation included overall adherence and subgroup analysis by gender, measured both before and 3 years after the index ACS. Patients were grouped based on the number of recommendations followed: ≤ 2, 3–4, and ≥ 5.

**Figure 2:**
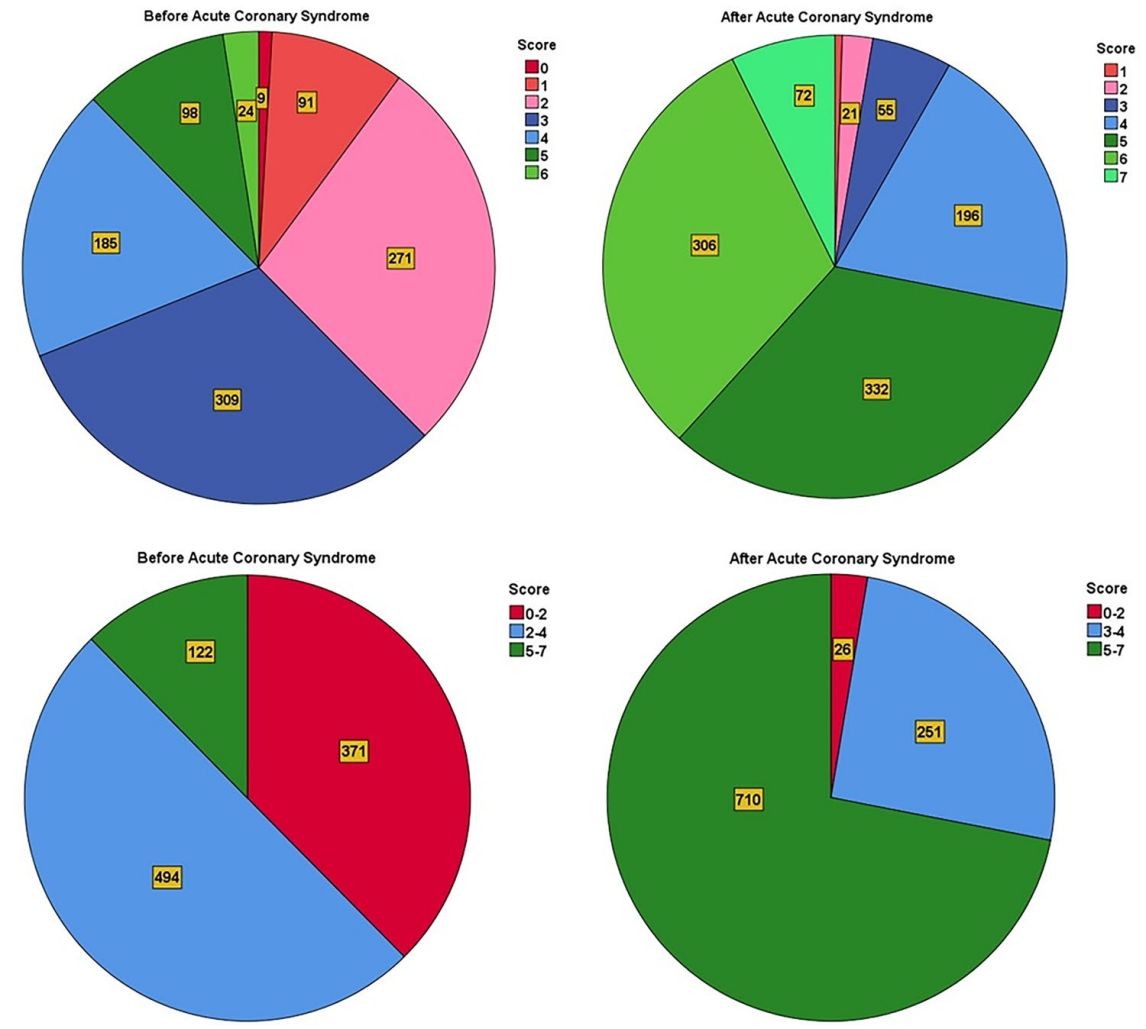
Comparison of adherence to secondary prevention guidelines before and 3 years after acute coronary syndrome.

Before ACS, 62.4% of patients adhered to three or more recommendations, increasing significantly to 97.4% by the 3rd‐year follow‐up (*p* < 0.001). Similarly, the percentage adhering to five or more recommendations rose from 12.4% before ACS to 71.9% at follow‐up (*p* < 0.001) (Figure 2).

**Figure 3:**
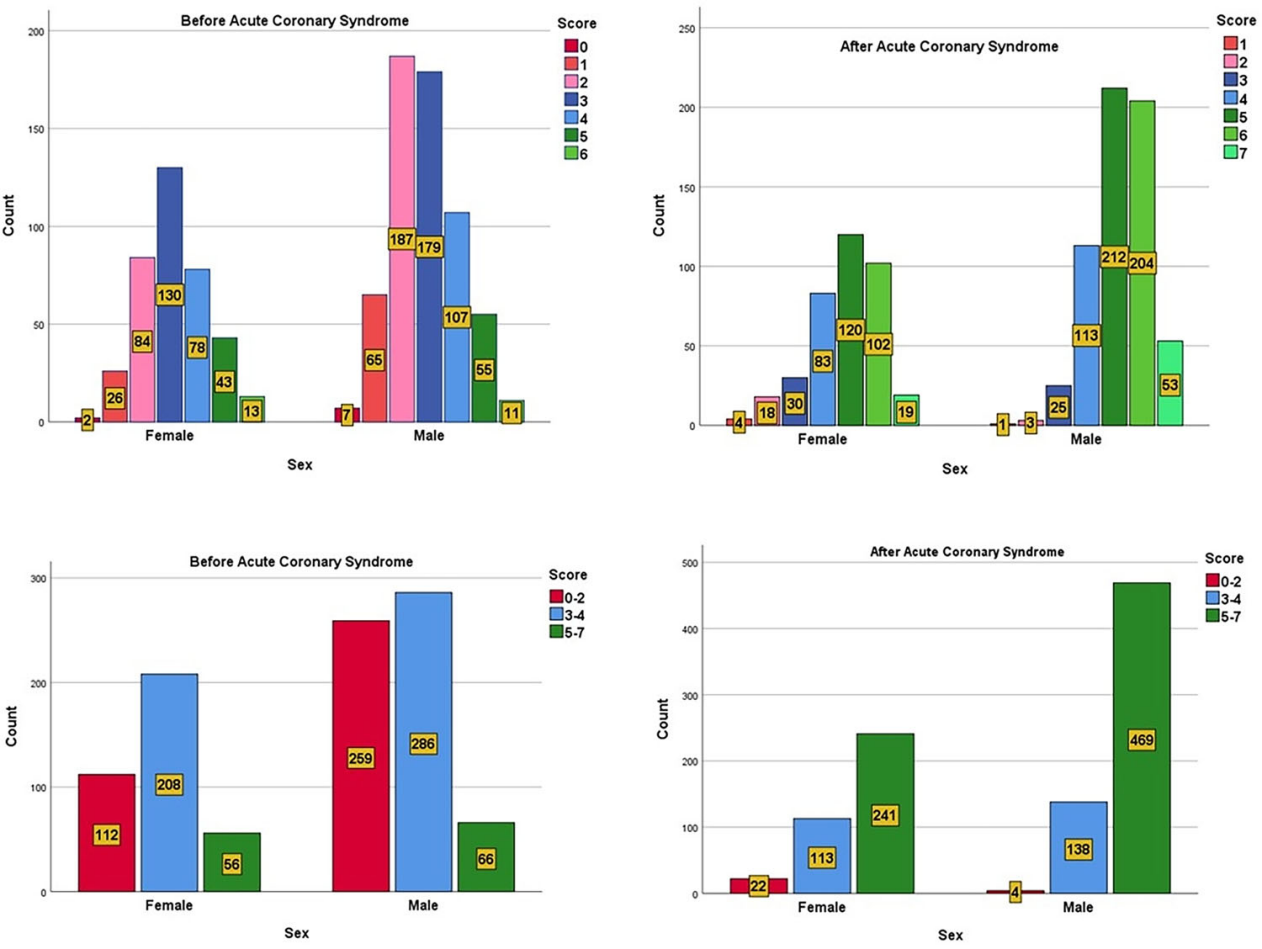
Gender‐specific comparison of adherence to secondary prevention guidelines before and 3 years after acute coronary syndrome.

A subgroup analysis was conducted to examine patient characteristics associated with better adherence. Female patients exhibited higher adherence rates before ACS, with 70.2% following at least three recommendations compared to 57.6% of males (*p* = 0.002). However, by the 3rd‐year follow‐up, adherence rates became comparable between genders, with 99.3% of males and 94.1% of females following at least three recommendations (*p* = 0.068) (Figure 3). Older patients (aged ≥ 65 years) demonstrated significantly higher adherence levels than younger patients, particularly for pharmacological therapies (*p* = 0.023). Additionally, patients with a history of prior cardiovascular events were more likely to follow guideline‐based recommendations compared to those with their first ACS event (*p* = 0.031). While socioeconomic status was not directly assessed, lower adherence rates were observed among patients requiring public healthcare assistance, indicating potential disparities in access to medications and follow‐up care.

By the end of the 3rd year, 406 patients (41.1%) experienced rehospitalization, and 115 patients (11.7%) had died. Cox regression analysis was conducted to identify demographic and clinical factors influencing rehospitalization and all‐cause mortality, as well as factors related to secondary prevention strategies. In the multivariate analysis, heart failure, anemia, elevated HbA1c levels, and reduced LVEF% were identified as significant predictors of rehospitalization (*p* = 0.041, *p* = 0.032, *p* = 0.029, and *p* = 0.015, respectively). Heart failure and elevated HbA1c levels were also associated with an increased risk of mortality (*p* = 0.044 and *p* = 0.015, respectively) (Table 3).

**Table 3:** Cox regression analysis of predictors for rehospitalization and mortality.

Patient characteristics	Univariate analysis			Multivariate analysis		
	HR	95% CI	*p*	HR	95% CI	*p*
*Rehospitalization*						
Age	0.930	0.792–1.068	0.19	1.007	0.995–1.020	0.27
Sex (male)	1.134	0.567–1.701	0.26	1.126	0.901–1.408	0.30
BMI	1.156	1.062–1.249	0.042	1.009	0.982–1.036	0.52
CRF	1.296	0.041–2.550	0.41	0.324	0.041–2.550	0.29
HF	1.328	1.075–1.581	0.007	1.258	1.083–1.432	0.041
HT	1.073	0.911–1.235	0.29	0.916	0.748–1.120	0.39
Hemoglobin	0.418	0.316–0.520	< 0.001	0.482	0.356–0.652	0.032
HbA1c	0.202	1.075–1.328	< 0.001	1.550	1.108–1.992	0.029
eGFR	0.358	0.168–0.763	0.008	0.999	0.995–1.003	0.62
LVEF	0.466	0.285–0.631	< 0.001	0.624	0.427–0.913	0.015
ACS type (STEMI)	1.898	1.450–2.345	0.018	1.018	0.812–1.277	0.87
*Total mortality*						
Age	2.556	0.299–4.820	0.14	0.981	0.957–1.005	0.13
Sex (male)	0.601	0.071–1.131	0.31	1.151	0.739–1.795	0.53
BMI	1.328	1.075–1.581	0.037	1.012	0.960–1.065	0.67
CRF	1.418	1.316–1.520	0.041	0.826	0.486–1.405	0.48
HF	1.656	1.330–1.981	< 0.001	1.239	1.004–1.473	0.044
HT	0.952	0.575–1.328	0.74	0.741	0.509–1.079	0.42
Hemoglobin	0.466	0.168–0.763	0.008	0.848	0.644–1.052	0.17
HbA1c	3.326	1.130–5.522	0.019	1.868	1.779–1.956	0.015
eGFR	0.812	0.552–1.072	0.24	1.002	0.884–1.018	0.32
LVEF	0.730	0.449–1.011	0.39	0.703	0.460–1.076	0.11
ACS type (STEMI)	1.577	1.135–2.019	< 0.001	1.440	0.950–2.183	0.09

Abbreviations: ACS, acute coronary syndrome; BMI, body mass index; CRF, chronic renal failure; GFR, glomerular filtration rate; HbA1c, hemoglobin A1c; HF, heart failure; HR, hazard ratio; HT, hypertension; LVEF, left ventricular ejection fraction; STEMI, ST‐elevation myocardial infarction.

Among prevention measures, statin use, blood pressure control, ACEi/ARB therapy, and SGLT‐2i therapy were associated with a reduced risk of rehospitalization (*p* = 0.047, *p* = 0.049, *p* = 0.028, and *p* = 0.033, respectively). Additionally, statin use, ACEi/ARB therapy, and SGLT‐2i therapy were linked to a reduced risk of mortality (*p* = 0.025, *p* = 0.042, and *p* = 0.014, respectively) (Table 4).

**Table 4:** Multivariate Cox regression analysis of factors associated with rehospitalization and mortality.

Patient characteristics	Multivariate Cox regression analysis					
	Rehospitalization			Total mortality		
	HR	95% CI	*p*	HR	95% CI	*p*
Antiplatelet	0.814	0.456–1.171	0.12	0.903	0.542–1.263	0.17
Statin	0.489	0.353–0.622	0.047	0.528	0.451–0.604	0.025
Blood pressure regulation	0.643	0.351–0.935	0.049	0.967	0.419–1.515	0.33
ACEi/ARB treatment	0.467	0.344–0.589	0.028	0.611	0.451–0.771	0.042
SGLT‐2i treatment	0.845	0.752–0.938	0.033	0.823	0.688–0.958	0.014
Not using/quitting smoking	0.914	0.375–1.452	0.90	0.864	0.472–1.256	0.43
Physically active	1.028	0.811–1.244	0.51	1.073	0.804–1.341	0.27

Abbreviations: ACEi/ARB, angiotensin‐converting enzyme inhibitors/angiotensin II receptor blockers; BMI, body mass index; LVEF, left ventricular ejection fraction.

**Figure 4:**
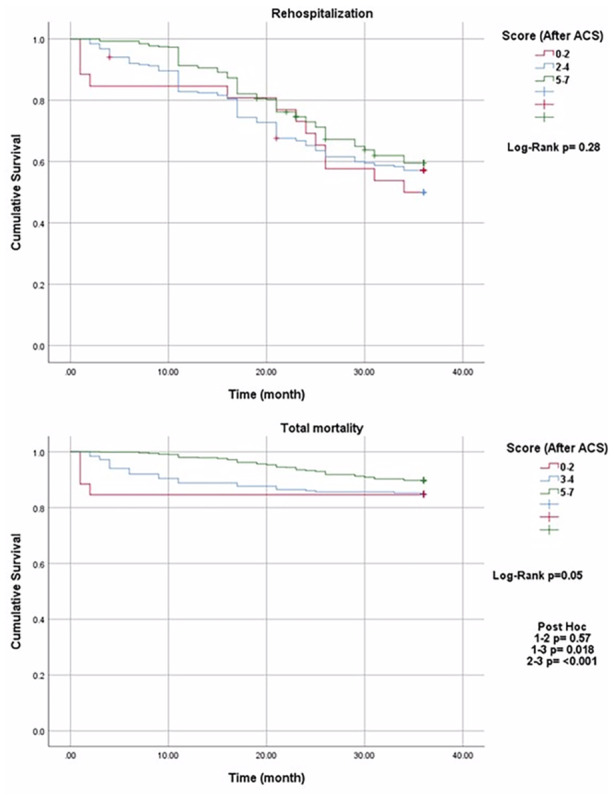
Comparison of rehospitalization and total mortality across recommendation implementation score groups over 3 years.

The frequency of rehospitalization and total mortality over 3 years was compared across secondary prevention strategy adherence score groups (0–2, 3–4, 5–7) using multiple regression analysis, with log‐rank tests conducted for further comparison. No significant difference was found between the groups in terms of rehospitalization (log‐rank *p* = 0.28). Regarding total mortality, no difference was observed between Groups 1 and 2 (*p* = 0.57), but Group 3 showed significantly lower mortality compared to both Groups 1 and 2 (*p* = 0.018 for Group 1 vs. Group 3 and *p* < 0.001 for Group 2 vs. Group 3) (Figure 4).

The results of this study demonstrate significant associations between adherence to guideline‐based secondary prevention strategies and improved clinical outcomes, including reduced rehospitalization and mortality rates. However, it is important to note that this is an observational study, and despite statistical adjustments for potential confounders, residual confounding may still influence the observed associations. As such, the findings should not be interpreted as establishing causality. The relationships identified between adherence and outcomes highlight the potential benefits of guideline‐directed therapies but underscore the need for further research, including randomized controlled trials, to confirm these findings and establish causal relationships.

## 4 Discussion

This study provides compelling evidence that adherence to guideline‐based secondary prevention strategies is associated with significantly better clinical outcomes in patients with both CAD and type 2 DM following an acute ACS event. Our findings demonstrate significant improvements in adherence to preventive strategies over 3 years, accompanied by reductions in rehospitalization and mortality. These results underscore the critical importance of promoting adherence to established guidelines to optimize outcomes in this high‐risk population [13, 14].

The baseline data revealed a concerning gap in preventive care, with fewer than two‐thirds of patients following three or more recommendations. This aligns with previous research showing poor adherence to guideline‐based therapies in real‐world settings, particularly in populations with complex comorbidities like CAD and DM [15, 16, 17]. Over the 3‐year follow‐up period, adherence rates improved markedly, with nearly all patients adhering to three or more recommendations and a significant proportion adhering to five or more. These findings mirror prior studies that have shown structured follow‐up and targeted interventions can substantially improve adherence rates over time [18, 19].

A critical aspect of this study was the explicit differentiation between true non‐adherence and clinically justified non‐use (e.g., contraindications or intolerance). This methodological distinction represents an important advance over previous studies that often conflate these categories, leading to potential misclassification bias. Our findings demonstrate that a significant proportion of patients who were not on guideline‐recommended therapies had valid clinical reasons for non‐use, such as contraindications or documented intolerance.

Our data reveal a striking “ACS effect” associated with adherence behaviors. Despite all patients having preexisting CAD, adherence improved significantly only after the ACS event. Several factors likely contributed to this change. First, the acute nature of ACS serves as a major psychological trigger, increasing patient awareness of disease severity and the potential consequences of nonadherence. Patients may be more receptive to medical advice and lifestyle changes following a life‐threatening event. Second, hospitalization for ACS often involves intensive patient education, closer medical supervision, and structured discharge planning, all of which reinforce adherence. Third, the increased frequency of follow‐up visits post‐ACS, combined with more aggressive treatment strategies and medication titration, may have contributed to better long‐term adherence. Lastly, healthcare providers may intensify efforts to optimize guideline‐directed medical therapy post‐ACS, recognizing the heightened risk of recurrent cardiovascular events. This “teachable moment” phenomenon suggests that healthcare systems should develop targeted interventions during this critical window when patients are most receptive to behavior change.

The gender‐specific analysis revealed baseline differences in adherence, with female patients showing higher adherence rates to preventive measures compared to males. However, by the 3‐year follow‐up, this difference was less pronounced, indicating that both genders benefited from improved adherence with consistent follow‐up care. This finding challenges common assumptions about gender disparities in cardiovascular care and suggests that structured follow‐up may help mitigate these differences. While gender disparities in adherence to cardiovascular therapies are well‐documented in the literature, our findings suggest that interventions targeting barriers to adherence can help minimize these gaps [20, 21].

The clinical impact of improved adherence was substantial and dose‐dependent. Improved adherence to preventive strategies was associated with significant reductions in rehospitalization and mortality, particularly among patients adhering to five or more recommendations. In multivariate analysis adjusted for potential confounders, statin use, blood pressure control, ACEi/ARB therapy, and SGLT‐2i therapy emerged as key contributors to reducing rehospitalization and mortality risks. Statin therapy was associated with reductions in both rehospitalization and mortality, consistent with evidence supporting its cardiovascular protective effects [22, 23, 24]. This aligns with recent findings by Chen and colleagues, who evaluated the incidence and predictors of statin adherence in a large cohort of patients with atherosclerotic cardiovascular disease, demonstrating that adherence to high‐intensity statin therapy was associated with a 38% reduction in MACE, though only 43.2% of eligible patients maintained optimal adherence at 2 years postinitiation [25]. Similarly, ACEi/ARB therapy, where indicated, and SGLT‐2i therapies were independently associated with lower mortality, corroborating findings from large‐scale trials such as EMPA‐REG OUTCOME and CANVAS, which demonstrated the benefits of SGLT‐2 inhibitors in reducing cardiovascular events and mortality in patients with diabetes and established cardiovascular disease [26, 27].

It is important to emphasize that our adherence assessment was indication‐specific, particularly for ACEi/ARB therapy. ACEi/ARB adherence should only be assessed in patients with appropriate indications, such as those with hypertension, heart failure, or chronic kidney disease. In this cohort, ACEi/ARB use was associated with improved outcomes, but this relationship is context‐dependent, emphasizing the need to consider patient‐specific factors, such as blood pressure status or heart failure, when evaluating adherence. In particular, ACEi/ARB therapy has well‐documented benefits in patients with heart failure or left ventricular dysfunction, reinforcing the importance of assessing adherence in these clinically relevant subgroups.

Our risk factor analysis identified several high‐risk clinical phenotypes that were associated with worse outcomes. Heart failure, elevated HbA1c levels, anemia, and reduced LVEF% were significant predictors of rehospitalization, consistent with prior studies emphasizing the prognostic value of these clinical parameters in patients with ACS [28, 29, 30, 31]. Similarly, heart failure and poor glycemic control were strongly associated with increased mortality. This suggests that risk stratification tools incorporating these variables could help identify patients requiring more intensive monitoring and management strategies. These findings underscore the importance of comprehensive management strategies addressing heart failure symptoms and glycemic control to improve outcomes in this patient population.

Despite the overall improvements in adherence and outcomes, adherence to certain preventive measures, such as smoking cessation and physical activity, showed minimal change over the follow‐up period. This highlights a critical gap in current secondary prevention strategies and suggests that standard counseling approaches may be insufficient for these behavioral domains. These findings highlight the persistent challenges in modifying lifestyle behaviors, which have been documented in prior research [32]. Our data support the need for novel approaches such as personalized counseling and behavioral therapies may be necessary to achieve meaningful improvements in these domains [33].

Our study primarily aimed to classify patients into adherence groups to assess the overall impact of adherence to preventive measures. However, identifying the independent contributions of each preventive measure in reducing mortality and rehospitalization could provide valuable insights for optimizing patient management. Further analyses are warranted to determine which of these measures exert the most significant influence on clinical outcomes.

Social determinants of health likely influenced our adherence patterns, though our study design limited exploration of these factors. While our study focused on adherence levels to seven preventive strategies, we acknowledge that socioeconomic and geographic factors may shape adherence patterns. Due to the retrospective design, an in‐depth exploration of these disparities was not feasible. Differences in adherence related to insurance status or healthcare setting could yield important insights; however, the lack of comprehensive socioeconomic data in our data set precluded a detailed subgroup analysis.

Moreover, adherence is influenced by multiple barriers, including medication costs, adverse effects, limited patient education, and psychological factors. The retrospective nature of our study prevented a systematic evaluation of these determinants. Given their potential impact on adherence, future prospective research incorporating patient‐reported challenges is essential to better elucidate and address adherence gaps.

A novel aspect of our analysis was exploring whether absolute adherence or improvement in adherence (delta) drives outcomes. A key aspect of this study is determining whether absolute post‐ACS adherence is the primary driver of outcomes or if the improvement (delta) from baseline also plays a role in risk reduction. While patients achieving high post‐ACS adherence demonstrated significantly better outcomes, our findings suggest that the degree of improvement in adherence following ACS may also be an influential factor in reducing adverse events. The Kaplan–Meier survival analysis (Figure 4) illustrates that patients with low adherence (Score 0–2) exhibited higher event rates early in the follow‐up, suggesting an initial vulnerability in this group. Over time, event‐free survival in this cohort stabilized, likely due to the attrition of high‐risk individuals experiencing early adverse events. This pattern highlights the potential role of adherence improvement (delta) as a determinant of outcomes, reinforcing the need for interventions that facilitate rapid adherence enhancement in the post‐ACS phase. This “delta effect” represents an important conceptual advance in understanding the temporal dynamics of secondary prevention. Future studies should further investigate whether the magnitude of adherence improvement independently contributes to long‐term risk reduction beyond absolute adherence levels.

Our study has several methodological strengths but also opportunities for enhancement in future research. While this study provides valuable insights into the relationship between adherence and outcomes in patients with CAD and DM following ACS, several methodological enhancements could strengthen future research in this area. First, incorporating objective measures of adherence, such as wearable device data for physical activity assessment and biochemical markers (e.g., exhaled carbon monoxide or cotinine levels) for smoking cessation verification, would improve the accuracy of adherence evaluation beyond self‐reported measures. Second, including beta‐blockers in the adherence scoring system would provide a more comprehensive assessment of guideline‐directed medical therapy, particularly given their Class I recommendation for post‐ACS patients despite potential concerns in diabetic populations. Third, a systematic evaluation of socioeconomic factors and their impact on adherence patterns would offer valuable insights into barriers to adherence and inform targeted interventions to address disparities in care.

The ideal future study design would be a prospective, multicenter randomized controlled trial comparing structured adherence‐enhancement interventions against standard care. To establish causal relationships between adherence and clinical outcomes, a prospective, multicenter randomized controlled trial comparing structured adherence‐enhancement interventions against standard care would be ideal. Such a study would not only address the limitations of observational design but also improve generalizability across diverse healthcare settings and patient populations. Additionally, longer follow‐up periods would allow for the assessment of cumulative benefits of sustained adherence and provide insights into the long‐term impact of various prevention strategies on cardiovascular outcomes in this high‐risk population.

The potential long‐term benefits of sustained adherence extend beyond our 3‐year follow‐up period. While this study focused on a 3‐year follow‐up period, the long‐term implications of sustained adherence to guideline‐based secondary prevention strategies warrant consideration. Evidence from large‐scale trials and longitudinal studies suggests that the benefits of adherence to therapies such as statins, ACEi/ARBs, and SGLT‐2 inhibitors may extend beyond the initial years post‐ACS, with cumulative effects on reducing cardiovascular events and improving survival. For instance, prolonged statin use has been associated with stabilization of atherosclerotic plaques and reduced progression of CAD, while SGLT‐2 inhibitors have demonstrated sustained benefits in heart failure prevention and renal protection. Similarly, lifestyle modifications, such as smoking cessation and regular physical activity, are likely to yield greater long‐term benefits, including reduced risks of recurrent ACS, stroke, and all‐cause mortality. These findings underscore the importance of not only initiating but also maintaining adherence to secondary prevention strategies over the long term.

A key strength of our retrospective approach was the ability to efficiently evaluate multiple guideline‐recommended strategies simultaneously and their associations with important clinical outcomes. This methodological choice was particularly appropriate given the urgent need for evidence to improve secondary prevention in this high‐risk population. While prospective studies offer certain advantages, the time‐sensitive nature of addressing suboptimal adherence in clinical practice justified our approach, which allowed for timely analysis of a comprehensive data set spanning 3 years of follow‐up. The insights generated can immediately inform quality improvement initiatives while more resource‐intensive prospective studies are being designed and implemented.

### 4.1 Limitations

Several important limitations should be considered when interpreting our findings. First, as an observational study, causality cannot be definitively established between adherence to preventive strategies and improved clinical outcomes. While efforts were made to control for confounding factors through multivariate analyses, residual confounding may still influence the observed associations. As such, the findings should not be interpreted as establishing causality. The relationships identified highlight the potential benefits of guideline‐directed therapies but underscore the need for further research, including randomized controlled trials, to confirm these findings and establish causal relationships.

Second, adherence was self‐reported in certain domains, such as lifestyle modifications, which may be subject to recall or social desirability bias. Self‐reported measures of smoking cessation and physical activity, in particular, may limit the accuracy of adherence assessments. While this is a common limitation in observational studies, incorporating objective validation methods—such as biochemical markers (e.g., exhaled carbon monoxide or cotinine levels for smoking cessation) and wearable devices (e.g., accelerometers or heart rate monitors for physical activity)—could provide more reliable and objective measures in future research.

Third, while the study population was followed for 3 years, longer‐term follow‐up would be necessary to fully assess the impact of adherence on outcomes such as cardiovascular mortality and major adverse cardiovascular events (MACE). Additionally, the study was conducted in a single healthcare setting, which may limit the generalizability of the findings to other populations or healthcare systems with different resources and clinical practices.

Fourth, certain preventive measures, such as dietary changes, were not systematically assessed due to the lack of standardized methods for quantifying dietary adherence in routine clinical practice. Future studies should consider incorporating validated dietary assessment tools to better evaluate the role of nutrition in secondary prevention.

Fifth, while the study focused on adherence to key secondary prevention strategies, it did not explore important patient‐related factors—such as health literacy, socioeconomic status, or access to care—that are known to influence both adherence and outcomes. Although lower adherence rates were observed among patients requiring public healthcare assistance, suggesting potential disparities, these factors were not systematically evaluated. Due to the retrospective design, an in‐depth exploration of these disparities was not feasible. Differences in adherence related to insurance status or healthcare setting could yield important insights; however, the lack of comprehensive socioeconomic data in our data set precluded a detailed subgroup analysis. Moreover, adherence is influenced by multiple barriers, including medication costs, adverse effects, limited patient education, and psychological factors. The retrospective nature of our study prevented a systematic evaluation of these determinants. Given their potential impact on adherence, future prospective research incorporating patient‐reported challenges is essential to better elucidate and address adherence gaps.

Sixth, ACEi/ARB adherence was assessed across the entire cohort. However, these therapies should only be considered in patients with appropriate clinical indications (e.g., hypertension, heart failure, or chronic kidney disease). Assessing adherence in patients without these conditions may overestimate non‐adherence and limit interpretability. Adherence to ACEi/ARB therapy should therefore be evaluated only in clinically relevant subgroups to avoid overgeneralization of the results.

Seventh, while SGLT‐2 inhibitors have shown benefits in patients with both type 2 diabetes and CAD, not all patients with diabetes have indications for these agents—particularly those without significant renal or cardiovascular complications. Classifying nonuse of SGLT‐2 inhibitors as nonadherence in such cases may overestimate true nonadherence rates.

Finally, the exclusion of beta‐blockers from the adherence scoring system should be considered a limitation. Although beta‐blockers are not specifically emphasized for diabetic patients due to a weaker association with glucose control and a potential link to new‐onset diabetes, they remain a Class I recommendation for post‐ACS management, as emphasized in current guidelines. Their exclusion may introduce bias in assessing the overall impact of guideline‐directed therapy in this population. This could affect the interpretation of adherence patterns and their relationship to outcomes, as beta‐blockers play a well‐established role in reducing cardiovascular events and mortality in post‐ACS patients.

In summary, while our findings suggest strong associations between adherence to guideline‐based secondary prevention strategies and improved clinical outcomes, these conclusions must be interpreted with caution. The observational design, reliance on self‐reported data, and selective evaluation of therapies may introduce biases or limit generalizability. Although the cohort size of 987 patients may be considered relatively modest, it represents a well‐defined, high‐risk population with dual diagnoses of CAD and DM. The statistically significant findings observed underscore the strength of the associations and the potential impact of adherence interventions even within a moderately sized cohort. Nevertheless, these findings provide valuable insights into the potential benefits of optimizing adherence among high‐risk patients with CAD and type 2 DM and support the need for further high‐quality interventional studies to validate these associations.

Despite these limitations, a retrospective cohort design was the most appropriate methodological approach for our research question and setting. The extensive follow‐up period required (3 years), the need to observe natural adherence patterns in a real‐world clinical environment, practical resource constraints, and ethical considerations regarding randomization to different levels of guideline‐directed care all favored a retrospective approach. Furthermore, this design allowed us to efficiently generate important hypothesis‐generating evidence from a robust sample size that can inform future prospective interventional studies targeted at improving adherence to the most impactful prevention strategies identified in our analysis.

### 4.2 Clinical Implications

Our findings have several important clinical implications that can guide patient care and healthcare policy. First, the observed “ACS effect” suggests that the immediate post‐ACS period represents a critical window for intervention. Healthcare systems should implement structured discharge protocols that include comprehensive medication reconciliation, personalized education about the importance of secondary prevention, and early follow‐up appointments (within 7–14 days) to reinforce adherence behaviors when patients are most receptive to change. This approach aligns with the concept of the “teachable moment” in behavioral medicine, where significant health events can catalyze sustained behavior change.

Second, our data support a tiered approach to post‐ACS follow‐up based on adherence risk profiles. Patients exhibiting poor baseline adherence, especially those with heart failure, elevated HbA1c levels, or reduced LVEF, should receive more intensive monitoring and support. This could include more frequent clinic visits, pharmacist‐led medication management, telehealth check‐ins, or involvement of case managers to address barriers to adherence. Our risk factor analysis provides a framework for identifying these high‐risk patients early in their post‐ACS course.

Third, the persistent challenges with smoking cessation and physical activity highlight the need for specialized behavioral interventions beyond standard counseling. Cardiac rehabilitation programs, which remain underutilized despite Class I recommendations, should be more systematically integrated into post‐ACS care pathways. For smoking cessation specifically, combining pharmacotherapy with behavioral support and using innovative digital health tools to provide ongoing reinforcement may yield better results than traditional approaches.

Fourth, our findings regarding medication adherence underscore the importance of simplifying therapeutic regimens when possible. The use of fixed‐dose combinations, once‐daily dosing schedules, and medication synchronization could reduce pill burden and improve adherence, particularly for patients requiring multiple agents. Additionally, clinicians should proactively address medication costs and insurance coverage during hospital discharge planning to mitigate financial barriers to adherence.

Fifth, the gender‐specific differences in baseline adherence that diminished over time suggest that targeted, gender‐sensitive approaches to education and support may help overcome initial disparities. Healthcare systems should evaluate their current protocols for potential gender bias and implement strategies to ensure equitable care delivery and outcomes.

Sixth, the observed relationship between SGLT‐2 inhibitor use and improved outcomes supports the earlier introduction of these agents in patients with CAD and type 2 DM, beyond their traditional role in glycemic control. Our findings align with recent guideline updates that emphasize the cardioprotective benefits of SGLT‐2 inhibitors and suggest that these agents should be considered core components of secondary prevention strategies in this population.

Finally, our data indicate that comprehensive adherence assessment should become a standard quality metric in cardiovascular care. Healthcare systems should develop standardized tools to measure adherence across multiple domains and implement systematic approaches to address identified gaps. Integrating adherence assessment into electronic health records could facilitate real‐time identification of nonadherence and trigger appropriate interventions.

## 5 Conclusion

This study highlights the critical role of adherence to guideline‐based secondary prevention strategies in improving long‐term outcomes in patients with CAD and DM after an ACS event. Over a 3‐year follow‐up period, adherence rates to key interventions, including antiplatelet therapy, statin use, blood pressure control, ACEİ/ARBs, and SGLT‐2i, improved significantly. Greater adherence was associated with a marked reduction in rehospitalization and all‐cause mortality, with adherence to five or more recommendations showing the most pronounced survival benefits. Furthermore, demographic and clinical factors such as heart failure, elevated HbA1c levels, and reduced LVEF were identified as significant predictors of adverse outcomes, reinforcing the need for targeted management strategies. These findings underscore the importance of optimizing adherence to preventive measures to mitigate risks and improve clinical outcomes in this high‐risk population, providing valuable insights for enhancing real‐world management practices.

## Author Contributions

Nur Kamer Kaya İnalkaç and İbrahim Keleş conceived of the presented idea. Nur Kamer Kaya İnalkaç and Fuat Polat developed the theory and performed the computations. Fuat Polat verified the analytical methods. İbrahim Keleş supervised the findings of this study. All authors discussed the results and contributed to the final manuscript.

## Ethics Statement

Cerrahpaşa Medical Faculty Clinical Research Ethics Committee approved the study protocol with the decision dated 02/08/2019 and numbered 30330229‐604.01.02‐118994. This study was conducted in accordance with the principles of the Declaration of Helsinki.

## Consent

All participants involved in this study provided informed consent before their inclusion. The consent process was conducted according to ethical standards, explaining the study's purpose, procedures, potential risks, and benefits. Participants were assured of confidentiality, voluntary participation, and the right to withdraw without consequences. Participants in this study were informed that the data collected may be used for publication purposes while ensuring anonymity and confidentiality. The consent for publication includes an understanding that no personally identifiable information will be disclosed. Written informed consent was obtained from all participants who participated in this study.

## Conflicts of Interest

The authors declare no conflicts of interest.

## Supporting information

APPENDIX.

## Data Availability

Data supporting the findings of this study are available from the corresponding author, upon reasonable request, via the e‐mail address drfuatpolat@gmail.com. All relevant data supporting the findings of this study are available upon request and will be provided by the corresponding author. For any inquiries related to the methodology or data analysis, please contact the corresponding author.
