# Supplementary material for: Impact of Adherence to Guideline‐Directed Prevention Strategies on Clinical Outcomes in Patients With Coronary Artery Disease and Diabetes Mellitus Following Acute Coronary Syndrome: A 3‐Year Cohort Study
Source: Clin Cardiol. 2025 Jun 2;48(6):e70164. doi: 10.1002/clc.70164 (PMC12130282; doi:10.1002/clc.70164)
Supplement: Supplementary file 1 — APPENDIX. [file CLC-48-e70164-s001.docx]

**APPENDIX: SUPPLEMENTARY METHODS**

**Detailed Adherence Measurement Methodology**

**Adherence Measurement Process**

The adherence measurement and data reconciliation process employed a standardized hierarchical approach to ensure consistency across multiple data sources, including prescription records, clinical documentation, and patient self-reports. Discrepancies were resolved by prioritizing objective data (e.g., prescription refills, laboratory values) over subjective reports, weighting multiple consistent data points, and favoring recent data over historical trends. Specific reconciliation methods were applied for medication adherence (e.g., using medication possession ratio ≥80%), blood pressure (e.g., mean readings over 3-month periods, prioritizing home or ambulatory monitoring), and lifestyle modifications (e.g., objective verification of smoking status, corroborating evidence for physical activity). In cases of equally valid contradictory data, the lower adherence level was conservatively assigned to avoid overestimation. All discrepancy resolutions were documented with rationale codes to ensure transparency and enable sensitivity analyses, maintaining a robust and consistent adherence classification system.

**Medical Record Analysis and Adherence Classification**

Medical records were systematically analyzed by two trained clinical researchers working independently, with discrepancies resolved by a third investigator. Inter-rater reliability was assessed using Cohen's kappa coefficient, which exceeded 0.85 for all adherence classifications, indicating excellent agreement. Electronic health records were accessed via the hospital's secure database system using a standardized data extraction protocol. For each patient, outpatient clinic notes, hospitalization records, medication lists, laboratory results, and pharmacy dispensing records over a three-year follow-up period were reviewed.

**Adherence Classification Criteria**

- Adherence: Consistent use of prescribed medications or lifestyle modifications per guideline recommendations, documented by:
  - Prescription refill rates ≥80%.
  - Documentation of medication use in ≥75% of clinical encounters.
  - Self-reported adherence during structured follow-up interviews without contradictory evidence.
- Non-Adherence: Failure to follow prescribed treatment recommendations without medical justification, identified by:
  - Prescription refill rates <80%.
  - Documentation of irregular or discontinued medication use in clinical notes.
  - Self-reported non-adherence during follow-up interviews.
  - Laboratory evidence of non-adherence (e.g., lipid profiles inconsistent with statin therapy).
- Intolerance: Adverse effects warranting discontinuation or dose reduction, verified by:
  - Physician documentation of specific medication-related adverse events.
  - Temporal relationship between medication initiation and symptom onset.
  - Resolution of symptoms upon dose reduction or discontinuation.
  - Failed rechallenge or dose titration attempts when clinically appropriate.
- Clinically Justified Non-Use: Absence of guideline-recommended medications due to valid contraindications or intolerance, determined by:
  - Documentation of contraindications (e.g., advanced chronic kidney disease for SGLT-2 inhibitors, active liver disease for statins).
  - Physician documentation of intolerance (e.g., statin-induced myopathy, ACE inhibitor-associated cough).
  - Absence of prescription despite clear indication but with documented clinical justification.

**Distinguishing Contraindications, Intolerance, and True Non-Adherence**

Analyzing adherence to guideline-based secondary prevention strategies requires differentiating between contraindications, intolerance, and true non-adherence, as these represent distinct scenarios influencing medication use:

- Contraindications: Medical conditions or clinical factors rendering a medication harmful or inappropriate, based on established evidence and guidelines. Examples:
  - Statins: Contraindicated in active liver disease or during pregnancy.
  - ACEi/ARBs: Contraindicated in patients with a history of angioedema or severe hyperkalemia.
  - SGLT-2 inhibitors: Avoided in advanced chronic kidney disease (e.g., eGFR below guideline thresholds).
- Intolerance: Adverse effects precluding continued use despite no absolute contraindication. Examples:
  - Statins: Persistent muscle symptoms (e.g., myalgia or myopathy) leading to discontinuation.
  - Antiplatelet therapy: Recurrent minor bleeding events (e.g., epistaxis or bruising) resulting in therapy cessation.
  - ACEi/ARBs: ACE inhibitor-associated cough necessitating a switch to ARBs.
- True Non-Adherence: Failure to initiate, continue, or appropriately use prescribed therapies without contraindications or documented intolerance. Contributing factors include:
  - Patient-Related: Forgetfulness, financial barriers, lack of understanding, or poor health literacy.
  - Healthcare System-Related: Limited access to medications, insufficient follow-up, or inconsistent provider-patient communication.
  - Medication-Related: Complex dosing regimens or high pill burdens.

By distinguishing these categories, the analysis avoids misclassifying adherence rates. Patients with contraindications or intolerance were not classified as non-compliant, as their non-use was clinically justified. Only those without contraindications or intolerance who did not follow prescribed treatments were classified as truly non-compliant.

**Criteria for Appropriate vs. Inappropriate Non-Use of Medications**

This study aimed to assess adherence to guideline-based secondary prevention strategies. However, it is critical to differentiate between appropriate and inappropriate non-use of medications across all drug classes included in the analysis.

- Inappropriate Non-Use: Refers to cases where a medication was not prescribed or discontinued despite clear clinical indications, without valid contraindications or documented intolerance. Examples:
  - Statins: Omission in patients eligible for high-intensity statin therapy without valid reasons such as severe adverse effects.
  - Antiplatelet therapy: Lack of initiation or continuation in patients with prior ACS and CAD, despite no contraindications like active bleeding.
  - ACEi/ARBs: Non-prescription in patients with hypertension, heart failure, or other guideline-based indications, without documented contraindications like hyperkalemia or angioedema.
  - SGLT-2 inhibitors: Not initiated in diabetic patients with CAD, despite their documented benefits and absence of contraindications like advanced CKD.
- Appropriate Non-Use: Defined as the absence of medication due to valid clinical considerations or contraindications, ensuring alignment with guideline recommendations. Examples:
  - Statins: Discontinued in cases of severe adverse effects, such as rhabdomyolysis or persistent myopathy.
  - Antiplatelet therapy: Withheld in patients with high bleeding risk or active gastrointestinal bleeding.
  - ACEi/ARBs: Avoided in cases of recurrent angioedema or hyperkalemia.
  - SGLT-2 inhibitors: Not prescribed for patients with contraindications like recurrent genital infections or significant renal impairment.

**Standardized Definitions of Adherence to Secondary Prevention Measures**

To ensure consistent evaluation across all participants, we established precise definitions for adherence to each of the seven guideline-recommended preventive measures:

1. Statin Therapy
   - Adherent: Documentation of continuous high-intensity statin use (atorvastatin 40-80 mg or rosuvastatin 20-40 mg daily) with ≥80% prescription fill rate over the follow-up period
   - Non-adherent: Prescription fill rate <80%, use of lower than recommended intensity without documented intolerance, or complete discontinuation without medical justification
2. Antiplatelet Therapy
   - Adherent: Continuous use of prescribed antiplatelet regimen (single or dual antiplatelet therapy based on clinical indication) with ≥80% prescription fill rate
   - Non-adherent: Premature discontinuation of recommended therapy, prescription fill rate <80%, or switching agents without clinical indication
3. Blood Pressure Control
   - Adherent: Achievement of target blood pressure (≤140/90 mmHg) in ≥70% of clinical measurements during the follow-up period
   - Non-adherent: Blood pressure exceeding target in >30% of measurements despite absence of documented resistant hypertension
4. ACE Inhibitors/ARBs
   - Adherent: Continuous use of prescribed ACEi/ARB with ≥80% prescription fill rate in patients with appropriate indications (hypertension, heart failure, albuminuria)
   - Non-adherent: Prescription fill rate <80%, unauthorized discontinuation, or failure to initiate when indicated
5. SGLT-2 Inhibitors
   - Adherent: Continuous use of prescribed SGLT-2 inhibitor with ≥80% prescription fill rate in eligible diabetic patients
   - Non-adherent: Prescription fill rate <80%, unauthorized discontinuation, or failure to initiate when indicated without contraindications
6. Smoking Cessation/Non-smoking
   - Adherent: Documented abstinence from smoking throughout follow-up for former smokers or continued non-smoking status for never-smokers
   - Non-adherent: Any reported smoking activity during follow-up period or positive carbon monoxide testing when performed
7. Physical Activity
   - Adherent: Documented engagement in ≥150 minutes of moderate-intensity aerobic physical activity weekly in ≥75% of follow-up assessments
   - Non-adherent: Failure to achieve recommended activity levels in >25% of follow-up assessments without documented physical limitations

**Guideline-Recommended Medical Therapy (GRMT)**

Adherence to GRMT was assessed based on the following recommendations:

- Statin Therapy: Initiated or maintained according to the 2019 European Society of Cardiology (ESC) Guidelines on Dyslipidaemias. High-intensity statins (atorvastatin 40-80 mg or rosuvastatin 20-40 mg) were used.
- Antiplatelet Therapy: Dual antiplatelet therapy (DAPT) was initiated as per the 2017 ESC Guidelines for the Management of Acute Myocardial Infarction in Patients Presenting with STEMI, using aspirin and a P2Y12 inhibitor (clopidogrel, ticagrelor, or prasugrel, depending on clinical indications and tolerance). Baseline antiplatelet use was assessed for all patients, as all participants had a prior CAD diagnosis, and antiplatelet therapy is a standard secondary prevention recommendation.
- Antihypertensive Therapy: Initiated or adjusted based on the 2018 ESC/European Society of Hypertension (ESH) Guidelines, targeting a blood pressure of ≤140/90 mmHg. ACE inhibitors (ACEi)/ARBs, calcium channel blockers (CCBs), and thiazide diuretics were first-line drugs, with beta-blockers, alpha-blockers, and mineralocorticoid receptor antagonists (MRAs) used as adjunctive therapy when indicated. Adherence to ACEi/ARB therapy was assessed only in patients with hypertension or relevant indications.
- SGLT-2 Inhibitors: Introduced in eligible patients with type 2 DM and CAD as recommended by the 2022 ESC Guidelines on Cardiovascular Disease Prevention. Empagliflozin and dapagliflozin were the primary agents used.

**Complete Inclusion and Exclusion Criteria**

Inclusion Criteria:

- Age ≥18 years
- Confirmed diagnosis of coronary artery disease (CAD) based on coronary angiography demonstrating ≥50% stenosis in at least one major epicardial coronary artery
- Established diagnosis of type 2 diabetes mellitus (defined as fasting plasma glucose ≥126 mg/dL, HbA1c ≥6.5%, or ongoing antidiabetic medication use with prior physician diagnosis)
- Presentation with acute coronary syndrome (ACS) during the study period (September 2019 to December 2022), defined by:
  - Elevated cardiac biomarkers (troponin I or T above the 99th percentile upper reference limit)
  - At least one of the following: ischemic symptoms, new significant ECG changes, imaging evidence of new loss of viable myocardium, or identification of intracoronary thrombus
- At least one documented medical encounter in our healthcare system within 12 months prior to the index ACS event
- Minimum of 36 months of follow-up data available after the index ACS event or until death if occurring earlier

Exclusion Criteria:

- Life expectancy <1 year due to non-cardiovascular causes (e.g., terminal malignancy)
- Inability to provide informed consent or participate in follow-up assessments
- Cognitive impairment preventing reliable assessment of adherence
- Transfer to another healthcare facility within 30 days of the index ACS without subsequent follow-up at our institution
- Participation in interventional clinical trials that could influence adherence to standard guideline-recommended therapies
- Type 1 diabetes mellitus
- Pregnancy during the study period
- End-stage kidney disease, defined as estimated glomerular filtration rate (eGFR) <15 mL/min/1.73m² (CKD stage 5) or requiring renal replacement therapy
- Patients receiving any form of dialysis (hemodialysis, peritoneal dialysis) or with history of kidney transplantation
- Severe hepatic dysfunction (Child-Pugh class C)
- History of solid organ transplantation
